# Time to diagnosis of nontuberculous mycobacterial pulmonary disease and longitudinal changes on CT before diagnosis

**DOI:** 10.1016/j.heliyon.2024.e30060

**Published:** 2024-04-23

**Authors:** Makoto Hayashi, Hiroyasu Takishima, Soma Kishino, Keitaro Kishi, Kenji Takano, Shogo Sakai, Yusuke Kakiuchi, Satoshi Matsukura

**Affiliations:** Department of Respiratory Medicine, Showa University Northern Yokohama Hospital, Japan

**Keywords:** *Mycobacterium avium-intracellulare* complex pulmonary disease (MAC-PD), Nontuberculous mycobacterial pulmonary disease (NTM-PD), Time to diagnosis, Duration for diagnosis, Chest CT, Cavitary lesions, Diagnostic delay, Predictors

## Abstract

**Background:**

The healthcare burden of nontuberculous mycobacterial pulmonary disease (NTM-PD) is increasing, but the diagnosis remains challenging and sometimes requires considerable time. This nested case-control study aims to clarify the time to diagnosis of NTM-PD, the factors that affect diagnosis and diagnostic delay, and changes in CT findings before diagnosis.

**Patients and methods:**

We retrospectively analyzed 187 patients suspected of having NTM-PD based on computed tomography (CT) findings at our institution between January 2019 and September 2020. We investigated the time to diagnosis of NTM-PD for all suspected and diagnosed patients. Multivariate analyses identified the factors affecting diagnosis and diagnostic delay over 6 months. We also evaluated longitudinal changes in CT findings during the observation period using CT scoring system.

**Results:**

The median times to diagnosis of NTM-PD were 71.8 months in all suspected patients and 3.2 months in only the diagnosed patients. Multivariable analysis showed that severity of the cavity domain of the CT score and *anti*-glycopeptidolipid (GPL)-core immunoglobulin A (IgA) antibody positivity were significantly associated with establishing the diagnosis. A low CT score in the cavity domain was a risk factor for delayed diagnosis. In patients with delayed diagnosis, the total CT score was less severe than that in the early diagnosis patients at their first visits; however, it had deteriorated prior to the diagnosis.

**Conclusion:**

The diagnosis of NTM-PD sometimes required several years, and the absence or mild cavitation predicted a diagnostic delay. Of concern, a delay in diagnosis can result in a delay in treatment.

## Highlights

• Median times to diagnosis of NTM-PD were 71.8 months in all suspected patients and 3.2 months in the diagnosed patients.
• Severity of cavitation and anti-GPL-core IgA antibody positivity were associated with establishing a diagnosis.
• The absence of or mild findings of cavitation caused a delay in diagnosis.
• CT findings had deteriorated prior to diagnosis in patients who required >6 months to be diagnosed.

## 1 Introduction

The incidence and prevalence of nontuberculous mycobacterial pulmonary disease (NTM-PD) are increasing, and the healthcare burden of this disease is becoming a concern [1,2]. While the prognosis and predictors, disease behavior over the natural course, and the effectiveness of specific treatment for NTM-PD have been elucidated over the last decade [[3], [4], [5], [6], [7], [8]], diagnosis of the disease remains challenging and often can take more than a few months [9]. The duration required for the diagnosis of NTM-PD and for radiographic changes to occur during the pre-diagnostic period remains unknown. We conducted this retrospective cohort study with a nested case-control analysis aiming to 1) reveal the time to diagnosis of NTM-PD, 2) identify the factors affecting diagnosis, and 3) identify the factors affecting diagnostic delay, and 4) evaluate radiographic changes before diagnosis.

## 2 Methods

### 2.1 Data collection and patient groups

**Fig. 1:**
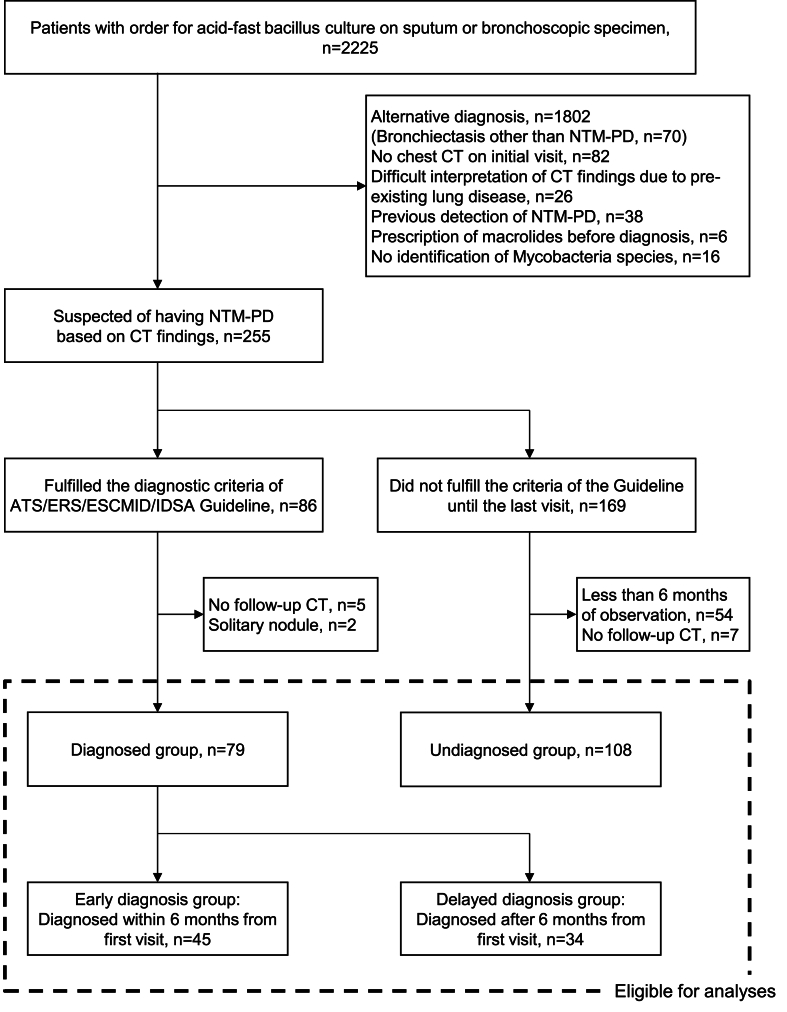
Patients eligible for analysis. Abbreviations: ATS American Thoracic Society; CT, computed tomography; ERS, European Respiratory Society; ESCMID, European Society of Clinical Microbiology and Infectious Diseases; IDSA, Infectious Diseases Society of America; NTM-PD, nontuberculous mycobacterial pulmonary disease.

We screened 2225 patients from whom acid-fast bacillus cultures of sputum or bronchoscopic specimens were ordered between January 2019 and September 2020 at Showa University Northern Yokohama Hospital, a teaching and referral hospital in Yokohama, Japan (Fig. 1). Among them, we identified 255 patients with suspected NTM-PD on chest computed tomography (CT) and retrospectively reviewed their electronic medical records. Two pulmonologists (MH and HT) evaluated the CT findings and made decisions through discussion in cases of disagreement. We collected data on the patients’ background, symptoms, CT findings, and results of bacteriological culture and blood tests including *anti*-glycopeptidolipid (GPL)-core immunoglobulin A (IgA) antibody dating back to their first visit. Acid-fast bacillus culture and chest CT findings were followed from the first visit until the end of observation. The patients were followed until they were diagnosed as having NTM-PD, or if no diagnosis was made, they were followed until their last visit before August 2023 and were censored.

Using the diagnostic criteria from the 2020 ATS/ERS/ESCMID/IDSA guideline [10], we analyzed the 187 patients suspected of having NTM-PD and classified them into the diagnosed group (n = 79) and undiagnosed group (n = 108) after excluding the following patients: 1) those with solitary nodule form of disease; 2) those who had no follow-up CT during the 6-month period before and after the time of diagnosis in the patients who required over 6 months to be diagnosed; and 3) the undiagnosed patients with a less than 6-month observation period or follow-up CT interval. Additionally, the diagnosed patients were divided into two groups: the early diagnosis group, comprising 45 patients who were diagnosed within 6 months of their first visit, and the delayed diagnosis group, comprising 34 patients who required more than 6 months to be diagnosed.

### 2.2 Classification and evaluation of CT findings

Disease forms on chest CT were classified as non-cavitary nodular/bronchiectatic (NB) disease, cavitary NB disease, fibrocavitary disease, and unclassifiable. CT findings were evaluated for severity at the first visit and within 6 months of the end of observation using a previously reported scoring system [11,12] in which points independently scored for the domains of bronchiectasis (maximum 9 points), cellular bronchiolitis (maximum 6 points), cavity (maximum 9 points), nodule (maximum 3 points), and consolidation (maximum 3 points) on chest CT are summed (maximum total 30 points).

### 2.3 Statistical analysis

Data are presented as numbers (percent) and median (interquartile range [IQR]). The cumulative diagnosis rate was estimated using the Kaplan-Meier method. Comparisons of characteristics or CT findings between each group were tested using the Chi-square test or Fisher's exact test and Wilcoxon signed-rank test for categorical and continuous variables, respectively. Steel's multiple comparison test was used in multiple comparisons. Multivariable logistic regression models were used to calculate odds ratios (ORs) for diagnosis and delayed diagnosis. Explanatory variables in the analysis for diagnosis were selected from previous studies [[13], [14], [15]] and also from the result of the above multivariable analysis for delayed diagnosis. In all analyses, we considered p < 0.05 as significant. All data were analyzed using JMP Pro v17.0.0 (SAS Institute Inc, Cary, NC, USA).

### 2.4 Ethical approval statement

The study protocol was in accordance with the Declaration of Helsinki and approved by the Ethical Committee of Showa University, Tokyo, Japan (approval no.: 2023-031-B) with waiver of the requirement for informed consent because of the retrospective study design.

## 3 Results

### 3.1 Time to diagnosis in all patients and diagnosed patients

**Fig. 2:**
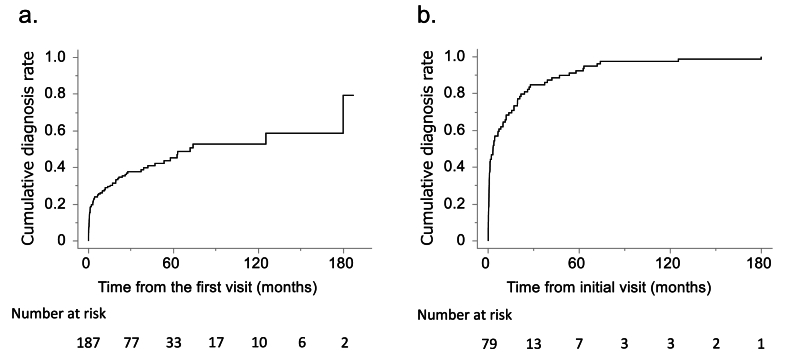
Cumulative diagnostic rates of nontuberculous mycobacterial pulmonary disease (NTM-PD). a. The cumulative diagnostic rate in all patients suspected of having NTM-PD. b. The cumulative diagnostic rate in only the patients diagnosed as having NTM-PD during the observation period.

Among all 187 patients with suspicion of NTM-PD, the median observation period was 23.2 (IQR 6.5–47.2) months and the median time to diagnosis was 71.8 (IQR 6.5–179.9) months. The cumulative rates of diagnosed patients were 24.1 % at 6 months, 29.1 % at 1 year, 35.5 % at 2 years, 45.3 % at 5 years, and 52.8 % at 10 years (Fig. 2a). For only the patients diagnosed as having NTM-PD, the median time to diagnosis was 3.2 (IQR 0.5–19.3) months. After 6 months of follow-up, 57.0 % of the patients had already been diagnosed, and that rate rose to 68.4 % at 1 year, 81.0 % at 2 years, and 92.4 % at 5 years (Fig. 2b).

### 3.2 Characteristics of the study patients and factors affecting diagnosis

Table S1 shows the characteristics of the diagnosed group and undiagnosed group. The most common causative NTM species was *Mycobacterium avium* (75.9 %), followed by *M. intracellulare* (20.3 %), *M. kansasii* (5.1 %), and *M. abscessus* complex (2.5 %). Acid-fast bacillus cultures were performed a median of 3 times in each group with no significant difference noted. There were no significant differences in patient background factors such as age, sex, body mass index, comorbidity, and concomitant use of immunosuppressive drugs between the two groups. The number of culture tests was not significantly different, although bronchoscopy was frequently performed in the diagnosed group (median 1, IQR 0–1 in the diagnosed group and median 0, IQR 0-0 in the undiagnosed group, p < 0.001). The CT findings were more severe in the diagnosed group; non-cavitary NB disease was less common (68 % and 96.3 %, p < 0.001), and severe CT scores were found for both the total score (median 11, IQR 8–14 and median 8, IQR 6–10, p < 0.001) and the score for every domain except nodule (bronchiectasis median 4, IQR 3–5 and median 3, IQR 3–4, p < 0.001; cellular bronchiolitis median 4, IQR 3–5 and median 3, IQR 3–4, p = 0.014; cavity median 0, IQR 0–4 and median 0, IQR 0-0, p < 0.001; consolidation median 1, IQR 1–2 and median 1, IQR 1–2, p = 0.026, respectively). In addition, *anti*-GPL core IgA antibody positivity was more common in the diagnosed patients (69.2 % and 35.8 %, p < 0.001).

We selected the explanatory variables for the multivariable logistic regression model from the factors reported to be of diagnostic utility using univariable logistic regression. The multivariable logistic regression model showed that the cavity domain of the CT score (aOR 155.732, 95 % CI 16.605–4308.802) and *anti*-GPL-core IgA antibody positivity (aOR 3.115, 95 % CI 1.341–7.488) were significant predictors for the diagnosis of NTM-PD (Table 1).

**Table 1:** Univariable and multivariable logistic regression model for diagnosis of NTM-PD.

	Univariable logistic regression			Multivariable logistic regression		
	Crude OR	95 % CI	p Value	Adjusted OR	95 % CI	p Value
Age, yrs	0.994	0.968–1.020	0.644			
Sex						
Female	reference					
Male	1.202	0.615–2.349	0.590			
BMI, kg/m2						
≥18.5	reference					
<18.5	1.250	0.620–2.520	0.533			
Unknown	1.300	0.566–2.985	0.536			
Symptomos						
Non-existence	reference					
Exsistence	0.690	0.380–1.253	0.223			
CT score						
Bronchiectasis	1.252	1.025–1.547	0.027^a^	0.763	0.065–9.256	0.830
Cellular bronchiolitis	1.286	1.017–1.639	0.038^a^	2.957	0.487–18.106	0.238
Cavity	1.970	1.420–0.508	<0.001^a^	155.732	16.605–4308.802	<0.001^a^
Nodule	1.674	0.975–2.907	0.063			
Consolidation	1.533	1.079–2.178	0.017^a^	1.785	0.280–11.735	0.540
*Anti*-GPL-Core IgA antibody						
<0.7U/ml	reference			reference		
≥0.7U/ml	4.031	1.863–8.725	<0.001^a^	3.115	1.341–7.488	0.008^a^
Unknown	1.770	0.834–3.754	0.137	1.774	0.784–4.130	0.170

Abbreviations: NTM-PD, nontuberculous mycobacterial pulmonary disease; OR, odds ratio; CI, confidence interval; BMI, body mass index; CT, computed tomography; GPL, glycopeptidolipid; IgA, immunoglobulin A.

a p < 0.05.

### 3.3 Characteristics of the diagnosed patients and the factor affecting time to diagnosis

The median observation periods in the early diagnosis group and the delayed diagnosis group were 0.7 (IQR 0.1–1.3) months and 21.2 (IQR 11.9–48.6) months, respectively. Between these groups, there were no significant differences in clinical background, but the number of acid-fast bacillus culture tests was higher in the delayed diagnosis group (Table 2). Regarding the CT findings, the delayed diagnosis group had significantly lower CT scores for total score (p = 0.002) and for each of the domains of cellular bronchiolitis (p = 0.009), cavity (p = 0.006), and consolidation (p = 0.005).

**Table 2:** Characteristics of the NTM-PD patients divided into early- and delayed-diagnosis groups.

	Early diagnosis		Delayed diagnosis	
Number of patients	45		34	p Value
Age, yrs	68 (58.5–76)		67 (54–73)	0.463
Female	33 (73.3)		25 (73.5)	0.984
BMI, kg/m^2^	19.3 (17.5–20.9)		19.6 (18.7–20.7)	0.498
	(n = 37)		(n = 29)	
Symptoms				
Symptom free	18 (40)		16 (47.1)	0.647
Cough	17 (37.8)		13 (38.2)	0.967
Sputum	17 (37.8)		10 (29.4)	0.603
Hemoptysis	8 (17.8)		4 (11.8)	0.540
Fever	3 (6.7)		0	0.255
Never smoker	27 (67.5)		23 (71.9)	0.689
Comorbidities				
Respiratory disease	6 (13.3)		6 (17.7)	1.000
Asthma	2 (4.4)		3 (8.8)	
COPD	1 (2.2)		1 (2.9)	
Cured tuberculosis	4 (8.9)		2 (5.9)	
Interstitial lung disease	0			
Lung cancer	0			
Non-respiratory disease	10 (22.2)		10 (29.4)	0.468
Sinusitis	2 (4.4)		0	
Collagen vascular disease	1 (2.2)		0	
Diabetes mellitus	5 (11.1)		3 (8.8)	
Cerebrovascular disease	2 (4.4)		0	
Heart disease	1 (2.2)		3 (8.8)	
GERD	1 (2.2)		3 (8.8)	
Inflammatory bowel disease	0		0	
Renal disease	1 (2.2)		1 (2.9)	
Concomitant drugs				
Inhaled corticosteroids	0		1 (2.9)	0.430
Systemic corticosteroids	1 (2.2)		2 (5.9)	0.574
Immunosuppressive agents	1 (2.2)		0	1.000
Biologic agents	0		0	
Number of culture tests	2 (2–3)		4 (3–7)	<0.001^a^
Expectorated sputum	2 (1–2.5)		4 (3–6)	<0.001^a^
Bronchoscopy	0 (0–1)		0 (0–1)	0.986
Diagnostic method				0.721
Expectorated sputum	26 (57.8)		21 (61.8)	
Bronchoscopy	19 (42.2)		13 (38.2)	
Mycobacterial species				
*Mycobacterium avium*	33 (73.3)		27 (79.4)	0.531
*M. intracellulare*	11 (24.4)		5 (14.7)	0.399
*M. kansasii*	3 (6.7)		1 (2.9)	0.630
*M. abscessus* complex	0		2 (5.9)	0.182
Disease form				
Non-cavitary NB disease	26 (57.8)		38 (82.4)	0.064
Cavitary NB disease	14 (31.1)		4 (11.8)	0.058
Fibrocavitary disease	5 (11.1)		2 (5.9)	0.692
Unclassifiable	0		0	
CT score				
Total	12 (9–15.5)		9 (6.8–11)	0.002^a^
Bronchiectasis	4 (3–5)		4 (3–5)	0.072
Cellular bronchiolitis	4 (3–5)		3 (3–5)	0.009^a^
Cavity	0 (0–5)		0 (0–0)	0.006^a^
Nodule	0 (0–1)		0.5 (0–1)	1.000
Consolidation	2 (1–2)		1 (1–2)	0.005^a^
*Anti*-GPL-Core IgA antibody (U/ml)				
≥0.7	23 (79.3)		13 (56.5)	0.077
	(n = 29)		(n = 23)	
*C*-reactive protein (mg/dl)	0.10 (0–0.50)		0.07 (0–0.14)	0.193
	(n = 44)		(n = 32)	
Serum albumin (mg/dl)	4.3 (3.7–4.4)		4.2 (4.1–4.3)	0.684
	(n = 42)		(n = 30)	

Abbreviations: NTM-PD, nontuberculous mycobacterial pulmonary disease; BMI, body mass index; COPD, chronic obstructive pulmonary disease; GERD, gastroesophageal reflux disease; NB, nodular/bronchiectatic; CT, computed tomography; GPL, glycopeptidolipid; IgA, immunoglobulin A.

Data are presented as number (%) or median (interquartile range).

Chi-square test or Fisher's exact test and Wilcoxon test were used for categorical and continuous variables, respectively.

a p < 0.05.

In the multivariable logistic regression model for delayed diagnosis (Table 3), only the cavity domain of the CT score was associated with a delayed diagnosis (aOR 0.735, 95 % CI 0.584–0.924). In the post hoc analysis to evaluate the effect of each radiographic finding contained in the CT score, the multivariate logistic regression model also showed that only the cavity domain was negatively correlated with the diagnostic delay (aOR 0.750, 95 % CI 0.571–0.947) (Table S2).

**Table 3:** Multivariable logistic regression model for delayed diagnosis of NTM-PD.

	Adjusted OR	95 % CI	p Value
CT score			
Cavity	0.735	0.584–0.924	0.008^a^
*Anti*-GPL-Core IgA antibody			
<0.7U/ml	reference		
≥0.7U/ml	0.338	0.092–1.244	0.103
Unknown	0.382	0.099–1.482	0.164

Abbreviations: NTM-PD, nontuberculous mycobacterial pulmonary disease; OR, odds ratio; CI, confidence interval; CT, computed tomography; GPL, glycopeptidolipid; IgA, immunoglobulin A.

a p < 0.05.

### 3.4 Longitudinal change in CT score

**Fig. 3:**
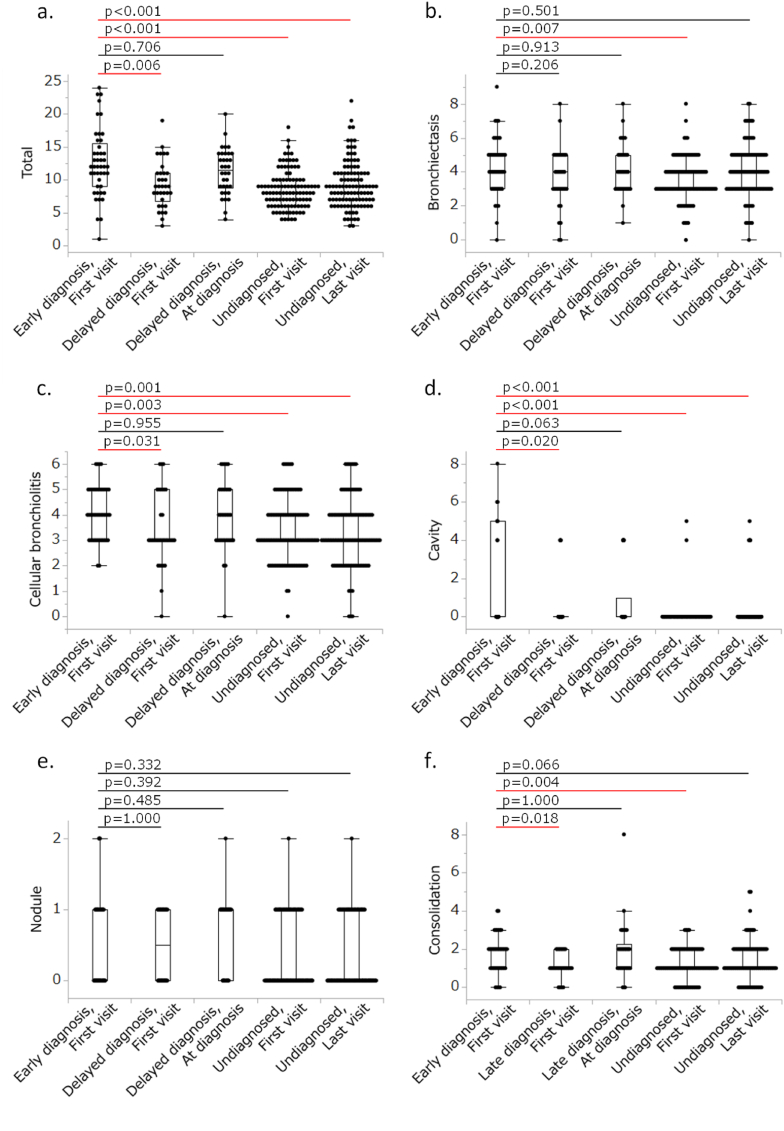
Comparisons of computed tomography (CT) scores for total score and each domain. The CT scores at the first visit of the early diagnosis group were used as the reference and were compared with the CT scores at the first visit and diagnosis of the delayed diagnosis group and the CT scores at the first visit and the end of observation of the undiagnosed patients. Steel's multiple comparison test was used for this multiple comparison. Red lines indicate p < 0.05. a. Total CT score. b. Bronchiectasis domain. c. Cellular bronchiolitis domain. d. Cavity domain. e. Nodule domain. f. Consolidation domain. (For interpretation of the references to colour in this figure legend, the reader is referred to the Web version of this article.)

Fig. 3a–f shows comparisons of the total CT score and that of each domain at the first visit and at the end of observation (“At diagnosis” or “Last visit” in the Figure) for the delayed diagnosis group and undiagnosed group, in reference to the CT scores at the first visit of the early diagnosis group. At the first visit, the total CT score in the delayed diagnosis group was significantly lower than that of the early diagnosis group (p = 0.006), whereas the difference was no longer significant at the time of diagnosis (p = 0.706). In contrast, the total CT score in the undiagnosed group was also significantly lower than that of the early diagnosis group; however, this difference remained significant until the end of the observation period (both, p < 0.001). Similar findings were observed for the cellular bronchiolitis and cavity domains.

Table 4 shows the total CT score and that for each domain at the first visit for the early diagnosis group and at the first visit and end of observation (“At diagnosis” or “Last visit” in the table) for the delayed diagnosis group and undiagnosed group. Within each of these groups, CT scores at the first visit and at the end of observation were compared. In the delayed diagnosis group, the total CT score and that for the consolidation domain showed significant increases during the observation period (p = 0.023 and p = 0.007, respectively). In contrast, the undiagnosed group did not show a significant change in total CT score (p = 0.191), whereas the scores deteriorated significantly for the bronchiectasis (p = 0.017) and cavity (p = 0.032) domains.

**Table 4:** CT scores in the early diagnosis group, delayed diagnosis group, and undiagnosed patients at the first visit and at the end of observation.

	Early diagnosis	Delayed diagnosis			Undiagnosed		
	First visit	First visit	At diagnosis	p Value	First visit	Last visit	p Value
Total	12 (9–15.5)	9 (6.8–11)	11.5 (8.8–14)	0.023^a^	8 (6–10)	9 (7–11)	0.191
Bronchiectasis	4 (3–5)	4 (3–5)	4 (3–5)	0.241	3 (3–4)	4 (3–5)	0.017^a^
Cellular bronchiolitis	4 (3–5)	3 (3–5)	4 (3–5)	0.062	3 (3–4)	3 (2–4)	0.689
Cavity	0 (0–5)	0 (0–0)	0 (0–1)	0.557	0 (0–0)	0 (0–0)	0.032^a^
Nodule	0 (0–1)	0.5 (0–1)	1 (0–1)	0.187	0 (0–1)	0 (0–1)	0.890
Consolidation	2 (1–2)	1 (1–2)	2 (1–2.3)	0.007^a^	1 (1–2)	1 (1–2)	0.277

Abbreviation: CT, computed tomography.

a p < 0.05.

## 4 Discussion

This study showed the time to diagnosis of NTM-PD to be a median of 71.8 months in all patients suspected of having NTM-PD and a median of 3.2 months in only the diagnosed patients. We also identified the cavity domain of the CT score and *anti*-GPL-core IgA antibody positivity as being associated with establishing a diagnosis. Furthermore, a low CT score for the cavity domain was a risk factor for delayed diagnosis, and the CT score deteriorated during the observation period in the delayed diagnosis group.

Diagnosis of NTM-PD is sometimes challenging because of the nonspecific and insidious nature of its radiologic findings or symptoms, or the difficulty in obtaining appropriate culture specimens due to the lack of sputum expectoration [16,17]; however, the time to diagnosis of NTM-PD is rarely reported [[18], [19], [20]]. One review article noted that the median time to diagnosis may be as high as 2 years or longer [18]. In contrast, one case study found that patients with a single detection of NTM in sputum had a mean time to diagnosis of 36 days [19]. Another study showed a median time from symptom onset to diagnosis of 10 months although it used an earlier version of diagnostic criteria issued in 1997 [20,21]. There is a wide range in the time to diagnosis between these reports. In the present study, the median time to diagnosis in all patients was longer than the above articles noted. There could be a selection bias related to the present study that extended the duration. Also, the heterogeneous background of the group could contribute to the extended duration. *Anti*-GPL-core antibody was positive in 35.8 % of the undiagnosed group. Given the high specificity of *anti*-GPL-core antibody [22], this would represent mild NTM-PD, which could not be diagnosed by current diagnostic criteria, or underdiagnosed active NTM-PD resulting from the infrequency of bronchoscopy. However, the lower prevalence of test-positive patients compared to the diagnosed group suggests more patients with etiologies other than NTM-PD. In the diagnosed patients, the median time to diagnosis was 3.2 months, which was within the range presented in previous studies. Meanwhile, 31.6 % remained undiagnosed after 1 year, indicating that sometimes considerable time is required to establish a diagnosis of NTM-PD. Previous reports suggested that the time to diagnosis depends on the factors such as strength of the physician's suspicion for NTM-PD and the quality of the bacteriology laboratory [18,20]. However, the radiographic differences between the early diagnosis group and delayed diagnosis group of the present study indicated that patient factors such as disease severity or bacterial burden may also affect the duration.

The multivariable analysis also showed that the cavity domain of the CT score and *anti*-GPL-core antibody positivity independently affected the diagnosis of NTM-PD. The presence of cavitary lesions is one of the well-known characteristics of NTM-PD [23,24] and is thus important in its diagnosis. The diagnostic performance of *anti*-GPL-core IgA antibody in *M. avium* complex pulmonary disease (MAC-PD) has been confirmed in previous studies [22,25]. It is known that the serum level of *anti*-GPL-core antibody is also elevated by *M. abscessus* complex infection [26]. In the present study, the distribution of the NTM species would be the reason why the *anti*-GPL-core antibody positivity predicted the diagnosis of NTM-PD overall.

The comparison between the early diagnosis group and delayed diagnosis group revealed significant differences in radiographic severity. Moreover, the multivariable analysis indicated that the cavity domain of the CT score was the predictor of the time to diagnosis. Griffith et al. found that decreasing the *M. avium-intracellulare* complex bacterial load during antibiotic therapy correlated with radiographic improvement [27]. Lee et al. described significant correlations between sputum smear positivity and radiographic findings using the scoring system we used [12]. In addition, the presence of cavity lesions is a predictor of disease progression and prognosis in MAC-PD [4,6]. These correlations support the hypothesis that the severity of radiographic findings reflects the bacterial load of NTM. On the basis of these findings, we suggest that even if NTM infection is truly present in patients with mild disease, it could not fulfill the diagnostic criteria due to low bacterial load at the first visit of the patients in the delayed diagnosis group.

In turn, we also investigated longitudinal changes in the CT findings. In the delayed diagnosis group, total CT score and those for the cellular bronchiolitis and cavity domains worsened longitudinally and became close to those in the early diagnosis group at the time of the diagnosis. Contrastingly, this trend did not appear in the undiagnosed group. The natural course of CT findings in patients with MAC-PD was previously reported to deteriorate in most patients without treatment [12,28]. Furthermore, a similar progressive trend was reported in patients before the diagnosis of *M. abscessus* complex-PD [29]. Our findings together with these other studies indicated that longitudinal radiographic deterioration could apply to patients with pulmonary infection caused by pathogenic NTM species prior to the diagnosis. Furthermore, it can be inferred that exceeding a certain threshold of radiographic findings would also surpass the threshold for mycobacteria detection by culture tests. Meanwhile, when we examined the changes in CT scores within each group, only the total score showed significant changes compatible with the comparisons among the different groups described above. This may be caused by insufficient statistical power due to the small number of study patients or insensitivity of the CT scoring system we used. Therefore, unfortunately, we could not provide specific radiographical features related to the diagnosis of NTM-PD, although the CT findings progressed on the whole.

The most important finding of this study is that the diagnosis of NTM-PD is often established during the follow-up period based on deterioration of the radiographic findings, even if the diagnosis is not made immediately after the initial examination. Meanwhile, early diagnosis and initiation of treatment before the progression of airway destruction may improve the prognosis of NTM-PD [30]; however, the sensitivity of the current diagnostic criteria in the early stage of the disease remains a concern.

This study has several limitations. This is a single-center, retrospective, observational study, and thus the population could be biased and was relatively small, which would affect the power and significance of the study. For the same reason, the duration of follow-up and the method of obtaining culture specimens were not standardized, which could result in an imprecise evaluation of the time to diagnosis. In addition, the follow-up period may not be long enough to diagnose subsequently developing disease, and thus some of the patients infected with NTM may remain undiagnosed. For these reasons, the results of the analyses may not be generalizable. Additionally, we could not evaluate the time between the establishment of the infection and the first visit in each patient, nor could we distinguish whether the NTM infection was present before or after the start of observation.

## 5 Conclusion

In the present study, the diagnosis of NTM-PD sometimes required several years, and the absence of or mild cavity lesions caused a delay in diagnosis. In the patients with delayed diagnosis, the CT findings deteriorated until the time of diagnosis. Of concern, a delay in diagnosis can result in a delay in treatment. Further development of techniques or methods with high sensitivity for detecting the early stage of the disease is desirable.

## CRediT authorship contribution statement

**Makoto Hayashi:** Writing – original draft, Visualization, Methodology, Investigation, Formal analysis, Data curation, Conceptualization. **Hiroyasu Takishima:** Writing – review & editing, Investigation, Data curation, Conceptualization. **Soma Kishino:** Writing – review & editing, Investigation, Data curation, Conceptualization. **Keitaro Kishi:** Writing – review & editing, Investigation, Data curation, Conceptualization. **Kenji Takano:** Writing – review & editing, Methodology, Formal analysis, Data curation, Conceptualization. **Shogo Sakai:** Writing – review & editing, Investigation, Conceptualization. **Yusuke Kakiuchi:** Writing – review & editing, Investigation, Data curation, Conceptualization. **Satoshi Matsukura:** Writing – review & editing, Supervision, Project administration, Conceptualization.

## Data availability statement

Question: Has data associated with your study been deposited into a publicly available repository?

Response: No. Data will be made available on request.

## Declaration of competing interest

The authors declare the following financial interests/personal relationships which may be considered as potential competing interests: Makoto Hayashi reports a relationship with Showa University Northern Yokohama Hospital that includes: speaking and lecture fees. If there are other authors, they declare that they have no known competing financial interests or personal relationships that could have appeared to influence the work reported in this paper.
