# Supplementary material for: Time to diagnosis of nontuberculous mycobacterial pulmonary disease and longitudinal changes on CT before diagnosis
Source: Heliyon. 2024 Apr 23;10(9):e30060. doi: 10.1016/j.heliyon.2024.e30060 (PMC11066632; doi:10.1016/j.heliyon.2024.e30060)
Supplement: Multimedia component 1 [file mmc1.docx]

| **Table S1**  Characteristics of the patients diagnosed as having NTM-PD and undiagnosed patients at first visits. | | | | | | | |
| --- | --- | --- | --- | --- | --- | --- | --- |
|  |  |  | Diagnosed group |  | Undiagnosed group |  |  |
| Number of patients | |  | 79 |  | 108 |  | p Value |
| Age, yrs | |  | 68 (57-75) |  | 68 (58-74.8) |  | 0.901 |
| Female |  |  | 58 (73.4) |  | 83 (76.9) |  | 0.590 |
| BMI, kg/m^2^ | |  | 19.5 (18.3-20.8)  (n=66) |  | 20.1 (18.3-21.4)  (n=93) |  | 0.309 |
| Symptoms | |  |  |  |  |  |  |
|  | Symptom free |  | 34 (43.0) |  | 37 (34.3) |  | 0.222 |
|  | Cough |  | 30 (38.0) |  | 48 (44.4) |  | 0.376 |
|  | Sputum |  | 27 (34.2) |  | 44 (40.7) |  | 0.361 |
|  | Hemoptysis |  | 12 (15.2) |  | 10 (9.3) |  | 0.214 |
|  | Fever |  | 3 (3.8) |  | 2 (1.9) |  | 0.415 |
| Never smoker | |  | 50 (69.4) |  | 70 (70.0) |  | 0.938 |
| Comorbidities | |  |  |  |  |  |  |
| Respiratory disease | |  | 12 (15.2) |  | 18 (16.7) |  | 0.786 |
|  | Asthma |  | 5 (6.3) |  | 9 (8.2) |  |  |
|  | COPD |  | 2 (2.5) |  | 2 (1.9) |  |  |
|  | Cured tuberculosis |  | 6 (7.6) |  | 5 (4.6) |  |  |
|  | Interstitial lung disease |  | 0 |  | 1 (0.9) |  |  |
|  | Lung cancer |  | 0 |  | 1 (0.9) |  |  |
| Non-respiratory disease | |  | 20 (25.3) |  | 24 (22.2) |  | 0.622 |
|  | Sinusitis |  | 2 (2.5) |  | 3 2.8) |  |  |
|  | Collagen vascular disease |  | 1 (1.3) |  | 1 (0.9) |  |  |
|  | Diabetes mellitus |  | 8 (10.1) |  | 4 (3.7) |  |  |
|  | Cerebrovascular disease |  | 2 (2.5) |  | 1 (0.9) |  |  |
|  | Heart disease |  | 4 (5.1) |  | 9 (8.3) |  |  |
|  | GERD |  | 4 (5.1) |  | 3 (2.8) |  |  |
|  | Inflammatory bowel disease | | 0 |  | 2 (1.9) |  |  |
|  | Renal disease |  | 2 (2.5) |  | 1 (0.9) |  |  |
| Concomitant drugs | |  |  |  |  |  |  |
|  | Inhaled corticosteroids |  | 1 (1.3) |  | 6 (5.6) |  | 0.127 |
|  | Systemic corticosteroids |  | 3 (3.8) |  | 0 |  | 0.074 |
|  | Immunosuppressive agents |  | 1 (1.3) |  | 2 (1.9) |  | 0.753 |
|  | Biologic agents |  | 0 |  | 1 (0.9) |  | 0.391 |
| Number of culture tests | |  | 3 (2-4) |  | 3 (1-5) |  | 0.658 |
|  | Expectorated sputum |  | 2 (1-4) |  | 3 (1-5) |  | 0.164 |
|  | Bronchoscopy |  | 0 (0-1) |  | 0 (0-0) |  | <0.001* |
| Diagnostic method | |  |  |  |  |  |  |
|  | Expectorated sputum |  | 47 (59.5) |  |  |  |  |
|  | Bronchoscopy |  | 32 (40.5) |  |  |  |  |
| Mycobacterial species | |  |  |  |  |  |  |
|  | *Mycobacterium avium* |  | 60 (75.9) |  |  |  |  |
|  | *M. intracellulare* |  | 16 (20.3) |  |  |  |  |
|  | *M. kansasii* |  | 4 (5.1) |  |  |  |  |
|  | *M. abscessus* complex |  | 2 (2.5) |  |  |  |  |
| Disease form | |  |  |  |  |  |  |
|  | Non-cavitary NB disease |  | 54 (68.4) |  | 104 (96.3) |  | <0.001* |
|  | Cavitary NB disease |  | 18 (22.8) |  | 2 (1.9) |  | <0.001* |
|  | Fibrocavitary disease |  | 7 (8.9) |  | 0 |  | 0.002* |
|  | Unclassifiable |  | 0 |  | 2 (1.9) |  | 0.509 |
| CT score | |  |  |  |  |  |  |
|  | Total |  | 11 (8-14) |  | 8 (6-10) |  | <0.001* |
|  | Bronchiectasis |  | 4 (3-5) |  | 3 (3-4) |  | 0.014* |
|  | Cellular bronchiolitis |  | 4 (3-5) |  | 3 (3-4) |  | 0.029* |
|  | Cavity |  | 0 (0-4) |  | 0 (0-0) |  | <0.001* |
|  | Nodule |  | 0 (0-1) |  | 0 (0-1) |  | 0.079 |
|  | Consolidation |  | 1 (1-2) |  | 1 (1-2) |  | 0.026* |
| Anti-GPL-Core IgA antibody (U/ml) | | |  |  |  |  |  |
|  | ≥0.7 |  | 36 (69.2)  (n=52) |  | 24 (35.8)  (n=67) |  | <0.001* |
| C-reactive protein (mg/dl) | |  | 0.07 (0-0.41)  (n=76) |  | 0.07 (0-0.28)  (n=98) |  | 0.466 |
| Serum albumin (mg/dl) | |  | 4.2 (3.9-4.4)  (n=72) |  | 4.2 (4.1-4.5)  (n=93) |  | 0.110 |
| Abbreviations: NTM-PD, nontuberculous mycobacterial pulmonary disease; BMI, body mass index; COPD, chronic obstructive pulmonary disease; GERD, gastroesophageal reflux disease; NB, nodular/bronchiectatic; CT, computed tomography; GPL, glycopeptidolipid; IgA, immunoglobulin A.  Data are presented as number (%) or median (interquartile range).  Chi-square test or Fisher’s exact test and Wilcoxon test were used for categorical and continuous variables, respectively.  *p<0.05. | | | | | | | |

| **Table S2** | |  |  |  |  |  |  |  |  |
| --- | --- | --- | --- | --- | --- | --- | --- | --- | --- |
| Univariable and multivariable logistic regression model for delayed diagnosis of NTM-PD. | | | | | | | | |  |
|  |  |  | Univariable logistic regression | | |  | Multivariable logistic regression | | |
|  |  |  | Crude OR | 95% CI | p Value |  | Adjusted OR | 95% CI | p Value |
| CT score | |  |  |  |  |  |  |  |  |
|  | Bronchiectasis |  | 0.782 | 0.581-1.023 | 0.084 |  |  |  |  |
|  | Cellular bronchiolitis | | 0.607 | 0.397-0.888 | 0.014* |  | 0.677 | 0.438-1.022 | 0.067 |
|  | Cavity |  | 0.735 | 0.572-0.907 | 0.008* |  | 0.750 | 0.571-0.947 | 0.023* |
|  | Nodule |  | 0.902 | 0.406-1.973 | 0.797 |  |  |  |  |
|  | Consolidation |  | 0.439 | 0.231-0.766 | 0.007* |  | 0.560 | 0.278-1.057 | 0.086 |
| Anti-GPL-Core IgA antibody | | | |  |  |  |  |  |  |
|  | <0.7U/ml |  | reference |  |  |  |  |  |  |
|  | ≥0.7U/ml |  | 0.339 | 0.095 | 0.077 |  |  |  |  |
|  | Unknown |  | 0.413 | 0.110 | 0.166 |  |  |  |  |
| Abbreviations: NTM-PD, nontuberculous mycobacterial pulmonary disease; OR, odds ratio; CI, confidence interval; CT, computed tomography; GPL, glycopeptidolipid; IgA, immunoglobulin A.  *p<0.05. | | | | | | | | | |
